# Combining deep learning with 3D stereophotogrammetry for craniosynostosis diagnosis

**DOI:** 10.1038/s41598-020-72143-y

**Published:** 2020-09-18

**Authors:** Guido de Jong, Elmar Bijlsma, Jene Meulstee, Myrte Wennen, Erik van Lindert, Thomas Maal, René Aquarius, Hans Delye

**Affiliations:** 1 Department of Neurosurgery, Radboudumc, Nijmegen, The Netherlands; 2 Technical Medicine, University of Twente, Enschede, The Netherlands; 3 Radboudumc 3D Lab, Radboudumc, Nijmegen, The Netherlands; 4 Department of Oral and Maxillofacial Surgery, Radboudumc, Nijmegen, The Netherlands

**Keywords:** Computational science, Medical imaging, Paediatrics

## Abstract

Craniosynostosis is a condition in which cranial sutures fuse prematurely, causing problems in normal brain and skull growth in infants. To limit the extent of cosmetic and functional problems, swift diagnosis is needed. The goal of this study is to investigate if a deep learning algorithm is capable of correctly classifying the head shape of infants as either healthy controls, or as one of the following three craniosynostosis subtypes; scaphocephaly, trigonocephaly or anterior plagiocephaly. In order to acquire cranial shape data, 3D stereophotographs were made during routine pre-operative appointments of scaphocephaly (n = 76), trigonocephaly (n = 40) and anterior plagiocephaly (n = 27) patients. 3D Stereophotographs of healthy infants (n = 53) were made between the age of 3–6 months. The cranial shape data was sampled and a deep learning network was used to classify the cranial shape data as either: healthy control, scaphocephaly patient, trigonocephaly patient or anterior plagiocephaly patient. For the training and testing of the deep learning network, a stratified tenfold cross validation was used. During testing 195 out of 196 3D stereophotographs (99.5%) were correctly classified. This study shows that trained deep learning algorithms, based on 3D stereophotographs, can discriminate between craniosynostosis subtypes and healthy controls with high accuracy.

## Introduction

Craniosynostosis is defined as the premature fusion of one or more cranial sutures. This results in cranial malformation and can lead to facial asymmetry, as well as functional consequences such as increased intracranial pressure, deafness, visual impairment and cognitive deficits^1,2^. The prevalence of isolated, non-syndromic craniosynostosis is 3.14 to 6 per 10,000 live births^3,4^. The three most common forms of isolated, non-syndromic craniosynostosis are scaphocephaly, trigonocephaly and anterior plagiocephaly which correspond to the premature fusion of the sagittal suture, the metopic suture, and a unilateral coronal suture, respectively^3,5–7^.

Early diagnosis and intervention is important as it often leads to more therapeutic options for surgeons and the best cosmetic results for patients^1,2^. Currently, computed tomography (CT) is the primary image technique used in craniosynostosis diagnosis^8,9^. CT is proven to be an accurate diagnostic tool in craniosynostosis, but exposes the infant to ionizing radiation, which can pose a health risk for radiation induced cancer^9–11^. Therefore a safer alternative diagnostic tool is needed.

Experts within tertiary healthcare centers can diagnose craniosynostosis quite accurately by visual examination of the shape of the head^8^. However, in the primary and secondary healthcare sectors, misdiagnosis of craniosynostosis still occurs due to the lack of expertise^12^. It therefore makes sense to study the potential of an imaging technique based on visual information: 3D stereophotogrammetry of the cranium. 3D stereophotogrammetry is a fast, radiation-free and patient-friendly method to evaluate the 3D morphology of the cranial shape^13^. 3D stereophotogrammetry has previously been used in combination with principal component analysis, a common machine learning technique, to characterize relevant aspects of the cranial shapes of trigonocephaly patients, scaphocephaly patients and healthy infants (controls)^14^. However, this technique cannot automatically classify these cranial shapes as scaphocephaly, trigonocephaly, anterior plagiocephaly or as a healthy child, which makes it irrelevant for clinical implementation.

To overcome this limitation, we suggest combining 3D stereophotogrammetry with the more modern machine learning technique ‘deep learning’. This facilitates direct classification of cranial shapes and makes clinical implementation more feasible. Deep learning has shown promising results in various fields of research, including medical image analysis^15–18^. Some benefits of deep learning prediction models are the possibility to evaluate complex patterns as well as non-linear patterns in data sets, effectively increasing the learning and classifying capacity of the model.

The goal of this study is to investigate if deep learning algorithms are capable to correctly classify the head shape of infants on 3D stereophotographs as healthy control or as a craniosynostosis patient with the accompanying subtype; scaphocephaly, trigonocephaly or anterior plagiocephaly.

## Results

The mean age at the acquisition of the 3D stereophotograph of the subjects was 5.1 months (SD: 3.0), 4.0 months (SD: 1.5), 6.9 months (SD: 4.7) and 4.6 months (SD: 1.6) for scaphocephaly patients, trigonocephaly patients, anterior plagiocephaly patients and healthy controls, respectively. There was a statistically significant difference between the group means as determined by one-way ANOVA(F(3,192) = 6.315, p < 0.001). Equal variances were not assumed based upon the Levene’s Test (p < 0.001). The Dunnett T3 post-hoc test showed a statistically significant difference between the mean ages of the trigonocephaly and plagiocephaly patients (p = 0.027). The differences between the mean ages of the trigonocephaly and scaphocephaly patients approached but did not reach statistical significant differences (p = 0.051).

### Deep learning network

Out of 196 patients and healthy controls, 195 subjects (99.5%) were correctly classified. One anterior plagiocephaly patient was classified as a healthy control (Table 1). Sensitivity and specificity was 100% in all cases except for plagiocephaly which had a sensitivity of 96.3% and the healthy cases which had a specificity of 99.2%.

**Table 1:**
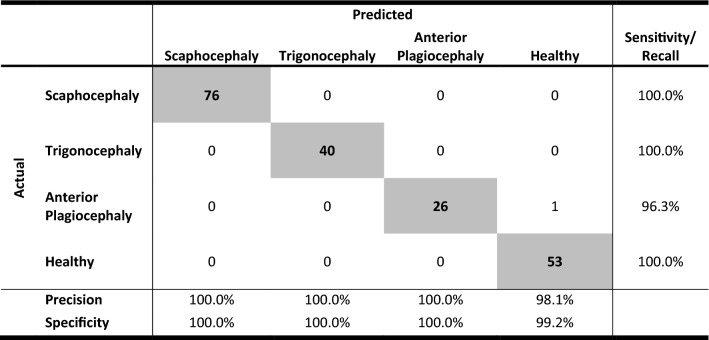
The confusion matrix of the test set with computed recall/sensitivity, precision and specificity.

## Discussion

In this study, we demonstrate that 3D stereophotogrammetry combined with deep learning can provide a basis to accurately classify cranial shapes of healthy controls, scaphocephaly patients, trigonocephaly patients and anterior plagiocephaly patients.

Our study is in line with results from a previous study, in which 3D stereophotographs were used to distinguish healthy subjects from subjects with scaphocephaly and trigonocephaly^14^. Both our study and the study of Meulstee et al. underline the feasibility of diagnosing craniosynostosis using 3D stereophotogrammetry, a non-invasive technique without the risk of exposing the patient to ionizing radiation. Other machine learning techniques have also shown the potential in the classification of (parts of) craniosynostosis on 3D stereophotographs with additional statistical analysis or preprocessing^19–21^.

Although the overall performance of the models presented in this study is good, there was one discrepancy between the prediction and the actual diagnosis. In the test dataset, one anterior plagiocephaly patient was classified as a healthy control. Anterior plagiocephaly cases were the least abundant within our dataset (n = 27), which means that the deep learning network had only a limited opportunity to learn the key features in the cranial shape associated with this craniosynostosis subtype. Furthermore, one of our experts reviewed the case and classified it as mild anterior plagiocephaly.

Deep learning algorithms tend to perform better when large datasets are used during training^17^. However, due to the low prevalence of isolated, non-syndromic craniosynostosis (3.14 to 6 per 10,000 live births)^3,4^, it is difficult to obtain large datasets for each craniosynostosis subtype. Smaller datasets can lead to suboptimal results because of overfitting, a process in which a network learns to recognize certain aspects specific to only the training cases, which negatively impacts the ability of the model to accurately classify new cases.

Several techniques have been used in this study to minimize the effect of overfitting. First, all 3D stereophotographs were sampled in the same orientation. This severely reduced heterogeneity and size of the data other than the actual shape differences. By removing this unwanted noise, neural networks need smaller databases to properly learn the key features of 3D stereophotographs to discriminate between the different subtypes of craniosynostosis. Second, there was an optimization in the number of vertices in the raycasting algorithm to minimize the amount of potential of overfitting features. Third, we applied feature scaling to correct for size differences and to amplify features of all subjects, which was desirable because of the differences in age between some of the groups. Fourth, we mirrored each 3D stereophotograph to decrease the impact of specific asymmetries in the collected cranial shapes, which can also lead to overfitting. Fifth, regularization techniques were used in the neural networks to further prevent overfitting. Finally, tenfold cross validation was used to determine the validity of each prediction model. The training and test results show a near identical distributions within the confusion matrices over all the folds suggesting good generalization of the model (Supplementary Table 1).

We applied a stratified tenfold cross validation setup without a separate test-set to determine the validity of each prediction model. Inclusion of a test-set could further establish the validity of a trained prediction model. Ideally a test-set should comprise a collection of externally collected 3D-stereophotographs and this could be the starting point for further research.

As mentioned before, larger datasets would be beneficial for craniosynostosis research. The rise of smartphone 3D stereophotogrammetry technology^22,23^ can aid in generating larger craniosynostosis datasets in the future. However, in the meantime data could be artificially generated based on the existing training data. This can be done by using a Generative Adversarial Network (GAN), which is another deep learning technique^24^. A GAN can generate data similar to the input of the network, thereby offering the possibility to synthetically enhance an existing dataset. This technique has previously been applied in medical images^25–28^ and also in improving accuracy in experiments with small-sized training datasets^27–30^. Due to the small size of the current 3D stereophotogrammetry dataset, GAN implementation could be of great value for future projects. The feasibility of the use of GANs in generating fictive datasets of trigonocephaly cases has been explored by our group but is not used within this study^31^.

3D photogrammetry is not the only radiation-free imaging modality that can be used to diagnose craniosynostosis as systematic physical examination, ultrasound and MRIcan also be utilized and have led to good results in the past^7,9,11,32,33^. It would be interesting to see if a deep learning algorithm would perform similarly when using images from another modality. Although each method has its own advantages, 3D stereophotogrammetry remains one of the fastest radiation-free methods for capturing the cranial shape for such diagnosis. Furthermore, 3D stereophotogrammetry technology is rapidly evolving, enabling the use smart-phone technology to make accurate 3D stereophotographs^22,23^.

In conclusion, this study shows that trained deep learning algorithms, based on 3D stereophotographs, can discriminate between craniosynostosis subtypes and healthy controls with high accuracy.

## Methods

### Data acquisition

A total of 160 CT-confirmed craniosynostosis patients and 53 healthy controls were retrospectively collected for this study. Healthy infants (controls) were selected based on their age (3 to 6 months old), which was similar to the age-range of the craniosynostosis patients. Some of the healthy controls have been previously included in a study for determining the normal evolution of the cranium in three dimensions^34^. 3D Stereophotogrammetry (3dMDCranial 3DMD, Atlanta, USA) with a five-pod configuration was used for image acquisition. 3D Stereophotographs were acquired by trained 3D photographers.

All craniosynostosis patients selected for this study were treated in the Radboudumc between July 2009 and September 2019 and diagnosed with isolated, non-syndromic premature closure of sutures, which was confirmed by CT as part of standard treatment protocol. Inclusion of patients was based on the availability of clinical pre-surgery 3D stereophotographs. We excluded 10 scaphocephaly, 4 trigonocephaly and 3 plagiocephaly patients due to the absence of a pre-surgery 3D stereophotograph or the presence of unremovable or unfixable imaging artefacts on the 3D stereophotograph. Unfixable imaging artefacts included the loss of anatomical landmarks or a large portion of the cranium. This resulted in the following craniosynostosis subtypes distribution in the included patients: scaphocephaly (n = 76), trigonocephaly (n = 40) and anterior plagiocephaly (n = 27). One 3D stereophotograph per patient or healthy reference was included.

The mean age and the standard deviation were computed for each group. A One-way ANOVA was performed to determine statistical significant differences between the mean ages of each group. A Levene’s test for homogeneity of variances was conducted. Finally a Dunnett T3 post-hoc test was performed to determine which groups were statistical significant different. Statistically significant differences were assumed at p < 0.05. All statistical data analyses were performed using SPSS version 25.0 (Armonk, NY: IBM Corp, 2017).

All research was performed in accordance with relevant guidelines and regulations. This study did not fall within the remit of the Medical Research Involving Human Subjects Act (WMO). This study was approved by the medical ethical review board of the Radboud University Medical Centre Nijmegen, The Netherlands (no. 2020-6128). The study has been reviewed by the ethics committee (Commissie Mensgebonden Onderzoek regio Arnhem—Nijmegen, Netherlands) on the basis of the Dutch Code of conduct for health research, the Dutch Code of conduct for responsible use, the Dutch Personal Data Protection Act and the Medical Treatment Agreement Act. Informed consent was waived by this same ethics committee (Commissie Mensgebonden Onderzoek regio Arnhem—Nijmegen, Netherlands). Furthermore, the 3D stereophotographs of the 53 healthy controls were collected as part of an ongoing program to form a large reference cohort for future studies and was approved by the medical ethical review board of the Radboud University Medical Centre Nijmegen, The Netherlands (no. 2018-4935).

### Data sampling

**Figure 1:**
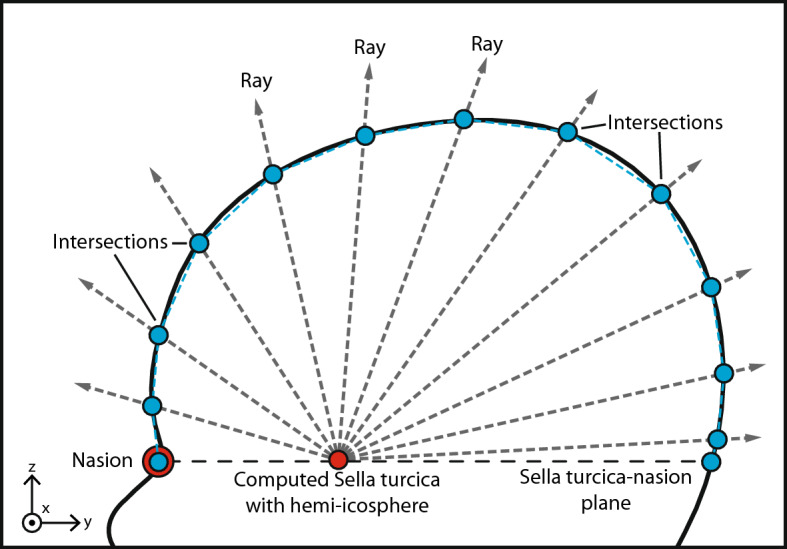
2D schematic representation of the head shape raycasting technique using a hemi-icosphere to determine the ray length from the sella turcica to the intersection of the outer surface of the 3D stereophotograph of the head.

Prior to data sampling, each 3D stereophotograph was manually positioned in the sella turcica-nasion orientation using the age specific computed cranial focal point^5,35^. Subsequently, data sampling was performed to ensure a standardized representation of each 3D stereophotograph. We used a raycasting algorithm for each 3D stereophotograph, utilizing a reference hemi-icosphere (r = 1 mm) consisting of 751 vertices. The center of the hemi-icosphere was placed on the location of the computed sella turcica and each ray was cast outward in the direction of each of the 751 vertices until the intersection with the 3D stereophotograph was reached (Fig. 1). The 751 vertices were the result of an optimization in which the raycasting algorithm was able to accurately capture the shape of the head while minimizing the amount of potential of overfitting features. Each raycast length, from the sella turcica to the intersection with the 3D stereophotograph, was stored for deep learning.

Because specific asymmetries in the collected cranial shapes can exist and can lead to potential underperformance of the deep learning model due to overfitting, data augmentation steps were used. A commonly used data augmentation step is image mirroring^36^. We mirrored each 3D stereophotograph over the mid-sagittal axis. Both the regular and the mirrored datasets were combined for deep learning model creation. Finally, for each ray the mean and standard deviation was computed over all the subjects. Each ray was then standardized by subtracting the per-ray mean and scaling using the per-ray unit variance^37^. This feature scaling technique was applied to correct for size differences and to amplify features of all subjects.

### Deep learning

We used a deep learning network with a categorical outcome, which classified the data of each subject as one of the following: healthy, scaphocephaly, trigonocephaly, anterior plagiocephaly. The used network was a conventional, feed-forward neural network holding respectively 192, 128, 64 and 32 nodes within the hidden layers. Activations within the hidden layers consisted of the Leaky Rectified Linear unit (Leaky ReLu), a variation on the Rectified Linear unit (ReLu)^38^, with an alpha of 0.2. The used regularization techniques were dropout^39^ (rate = 0.5), batch normalization^40^ (momentum = 0.8) and added gaussian noise (std = 0.5) on the input during training. The output layer had a softmax activation function with 4 nodes for healthy, scaphocephaly, trigonocephaly and anterior plagiocephaly. Training was performed using the Adam optimizer^41^ with a learning rate of 1 * 10^–3^, a decay of 1 * 10^–6^ and a clip/gradient normalization of 0.001. Batch sizes during training consisted of 256 samples and training was performed for up to 1,000 epochs. The categorical cross entropy is evaluated for the validation set and used as a stopping criterium. If no improvement of the validation categorical cross entropy was found for 50 epochs training was halted.

**Figure 2:**
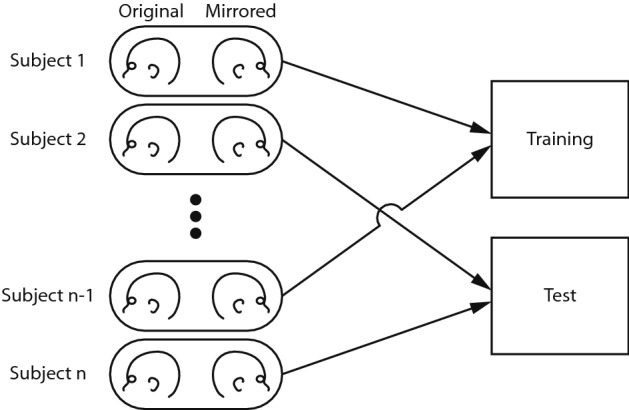
A subject’s original 3D stereophotograph and its mirrored counterpart stay linked throughout training and testing of the deep learning network to prevent cross-over.

For the training and testing of the deep learning network, a stratified tenfold cross validation was used. A subject’s original 3D stereophotograph and its mirrored counterpart stayed linked throughout training and testing of the deep learning network (Fig. 2). This ensures that one subject is only present in either the training or test dataset to preventing cross-over and misleading outcomes of the deep learning model.

The outcome of the deep learning network was a confusion matrix of the test dataset. Furthermore, the recall and sensitivity (true positive/(true positive + false negative)), precision (true positive/(true positive + false positive)) and specificity (true negative/(true negative + false positive) were computed based on the confusion matrix.

The software used for the deep learning network creation was Keras^42^ with the Tensorflow^43^ backend. The software used for statistical analysis of the deep learning results and preprocessing of the data was Scikit-learn^44^.

## Supplementary information

Supplementary Information 1.

## Data Availability

Data and algorithms available from the Radboud University Medical Center. Researchers who want to request data may apply to the corresponding author, who will discuss possibilities regarding data sharing with the local ethics committee. The algorithms and trained deep learning models used in the deep learning step can be applied for at the corresponding author.

## References

[1] Bristol RE, Lekovic GP, Rekate HL (2004). The effects of craniosynostosis on the brain with respect to intracranial pressure. Semin. Pediatr. Neurol..

[2] Delashaw JB, Persing JA, Broaddus WC, Jane JA (1989). Cranial vault growth in craniosynostosis. J. Neurosurg..

[3] Shuper A, Merlob P, Grunebaum M, Reisner SH (1985). The incidence of isolated craniosynostosis in the newborn infant. Arch. Pediatr. Adolesc. Med..

[4] Lee HQ (2012). Changing epidemiology of nonsyndromic craniosynostosis and revisiting the risk. J. Craniofac. Surg..

[5] de Jong GA, Maal TJJ, Delye H (2015). The computed cranial focal point. J. Cranio-Maxillofacial Surg..

[6] Krakow D (2017). Craniosynostosis. Obstet. Imaging Fetal Diagnosis Care Second Ed..

[7] Cunningham ML, Heike CL (2007). Evaluation of the infant with an abnormal skull shape. Curr. Opin. Pediatr..

[8] Fearon JA, Beals SP, Yu JC (2007). The diagnosis and treatment of single-sutural synostoses: Are computed tomographic scans necessary?. Plast. Reconstr. Surg..

[9] Kim HJ, Roh HG, Lee IW (2016). Craniosynostosis: Updates in radiologic diagnosis. J. Korean Neurosurg. Soc..

[10] Sheppard JP (2018). Risk of brain tumor induction from pediatric head CT procedures: A systematic literature review. Brain Tumor Res. Treat..

[11] Schweitzer T (2012). Avoiding CT scans in children with single-suture craniosynostosis. Child’s Nerv. Syst..

[12] Mathijssen IMJ (2015). Guideline for care of patients with the diagnoses of craniosynostosis: Working group on craniosynostosis. J. Craniofacial Surg..

[13] Heike, C. L., Upson, K., Stuhaug, E. & Weinberg, S. M. 3D digital stereophotogrammetry: A practical guide to facial image acquisition. *Head Face Med.* **6**, 18 (2010). 10.1186/1746-160X-6-18 PMC2920242 20667081

[14] Meulstee JW (2017). A new method for three-dimensional evaluation of the cranial shape and the automatic identification of craniosynostosis using 3D stereophotogrammetry. Int. J. Oral Maxillofac. Surg..

[15] Litjens G (2017). A survey on deep learning in medical image analysis. Med. Image Anal..

[16] Shen D, Wu G, Suk H-I (2017). Deep learning in medical image analysis. Annu. Rev. Biomed. Eng..

[17] Esteva A (2017). Dermatologist-level classification of skin cancer with deep neural networks. Nature.

[18] Lecun Y, Bengio Y, Hinton G (2015). Deep learning. Nature.

[19] Cho MJ, Hallac RR, Effendi M, Seaward JR, Kane AA (2018). Comparison of an unsupervised machine learning algorithm and surgeon diagnosis in the clinical differentiation of metopic craniosynostosis and benign metopic ridge. Sci. Rep..

[20] Porras AR (2019). Quantification of head shape from three-dimensional photography for presurgical and postsurgical evaluation of craniosynostosis. Plast. Reconstr. Surg..

[21] Bhalodia R (2020). Quantifying the severity of metopic craniosynostosis. J. Craniofac. Surg..

[22] Barbero-García I, Lerma JL, Miranda P, Marqués-Mateu Á (2019). Smartphone-based photogrammetric 3D modelling assessment by comparison with radiological medical imaging for cranial deformation analysis. Meas. J. Int. Meas. Confed..

[23] Barbero-García I, Lerma JL, Marqués-Mateu Á, Miranda P (2017). Low-cost smartphone-based photogrammetry for the analysis of cranial deformation in infants. World Neurosurg..

[24] Goodfellow, I. J. *et al.* Generative adversarial networks. arXiv preprint, arXiv:1406.2661 (2014).

[25] Wolterink, J. M., Kamnitsas, K. & Ledig, C. Generative adversarial networks and adversarial methods in biomedical image analysis. arXiv preprint, arXiv:1810.10352. 1–29 (2018).

[26] Kazeminia S (2020). Low-cost smartphone-based photogrammetry for the analysis of cranial deformation in infants. Artif Intell Med..

[27] Bailo, O., Ham, D. & Shin, Y. M. Red blood cell image generation for data augmentation using Conditional Generative Adversarial Networks. arXiv preprint, arXiv:1901.06219 (2019).

[28] Shin, H. *et al.* Medical image synthesis for data augmentation and anonymization using generative adversarial networks. In *Simulation and Synthesis in Medical Imaging. SASHIMI 2018. Lecture Notes in Computer Science*, Vol. 11037 (eds Gooya, A. *et al.*) (Springer, Cham, 2018). 10.1007/978-3-030-00536-8_1.

[29] Calimeri, F., Marzullo, A., Stamile, C. & Terracina, G. *Biomedical Data Augmentation Using Generative Adversarial Neural Networks*. (2017). 10.1007/978-3-319-68612-7_71.

[30] Bowles, C. *et al.* GAN Augmentation: Augmenting Training Data using Generative Adversarial Networks. arXiv preprint, arXiv:1810.10863 (2018).

[31] Sterkenburg A., de Jong G., Meulstee J., D. H. ISCFS 2019 Abstract Supplement 19-3-240|CRANIOSYNOSTOSIS/METOPIC|Generating fictive trigonocephaly data using a generative adversarial network to produce data to train deep learning algorithms. In *Plastic and Reconstructive Surgery—Global Open*, Vol. 7, (2019).

[32] Rozovsky K (2016). Cranial ultrasound as a first-line imaging examination for craniosynostosis. Pediatrics.

[33] Eley KA, Watt-Smith SR, Sheerin F, Golding SJ (2014). “Black Bone” MRI: A potential alternative to CT with three-dimensional reconstruction of the craniofacial skeleton in the diagnosis of craniosynostosis. Eur. Radiol..

[34] Meulstee JW (2019). The normal evolution of the cranium in three dimensions. Int. J. Oral Maxillofac. Surg..

[35] de Jong G (2017). Radiation-free 3D head shape and volume evaluation after endoscopically assisted strip craniectomy followed by helmet therapy for trigonocephaly. J. Cranio-Maxillofacial Surg..

[36] Shorten C, Khoshgoftaar TM (2019). A survey on image data augmentation for deep learning. J. Big Data.

[37] Jain AK, Dubes RC (1988). Algorithms for Clustering Data.

[38] Nair, V. & Hinton, G. E. Rectified linear units improve restricted boltzmann machines. In *Proceedings of the 27th International Conference on Machine Learning (ICML-10), June 21–24, 2010, Haifa, Israel* (eds. Fürnkranz, J. & Joachims, T.) 807–814 (Omnipress, 2010).

[39] Srivastava N, Hinton G, Krizhevsky A, Sutskever I, Salakhutdinov R (2014). Dropout: A simple way to prevent neural networks from overfitting. J. Mach. Learn. Res..

[40] Ioffe, S. & Szegedy, C. Batch normalization: Accelerating deep network training by reducing internal covariate shift. *32nd Int. Conf. Mach. Learn. ICML 2015* Vol. 1, 448–456 (2015).

[41] Kingma, D. P. & Ba, J. Adam: A Method for Stochastic Optimization. 1–15 (2014). 10.1145/1830483.1830503.

[42] Chollet, F. & others. Keras. (2015).

[43] Abadi, M. *et al.* TensorFlow: A system for large-scale machine learning TensorFlow: A system for large-scale machine learning. In *12th USENIX Symp. Oper. Syst. Des. Implement. (OSDI ’16)* 265–284 (2016). 10.1038/nn.3331.

[44] Pedregosa F (2011). Scikit-learn: Machine learning in {P}ython. J. Mach. Learn. Res..

