# Supplementary material for: Combining deep learning with 3D stereophotogrammetry for craniosynostosis diagnosis
Source: Sci Rep. 2020 Sep 18;10:15346. doi: 10.1038/s41598-020-72143-y (PMC7501225; doi:10.1038/s41598-020-72143-y)
Supplement: Supplementary file 1 — Supplementary Information 1. [file 41598_2020_72143_MOESM1_ESM.docx]

**Title**

Combining deep learning with 3D stereophotogrammetry for craniosynostosis diagnosis

**Authors**

Guido de Jong^1^ *

Elmar Bijlsma^1^

Jene Meulstee^3,4^

Myrte Wennen^1,2^

Erik van Lindert^1^

Thomas Maal^3,4^

René Aquarius^1^

Hans Delye^1^

**Affiliations**

^1^ Department of Neurosurgery, Radboudumc, Nijmegen, The Netherlands

^2^ Technical Medicine, University of Twente, Enschede, The Netherlands

^3^ Radboudumc 3D Lab, Radboudumc, Nijmegen, The Netherlands

^4^ Department of Oral and Maxillofacial Surgery, Radboudumc, The Netherlands

* corresponding author (Guido.deJong@radboudumc.nl)

# Supplementary Table 1

Supplementary Table 1: The confusion matrix of the training sets with computed recall/sensitivity, precision and specificity. Due to the use of the 10-fold cross-validation method, training numbers are 9 times higher than the number of patients.

|  |  | **Predicted** | | | |  | |  |
| --- | --- | --- | --- | --- | --- | --- | --- | --- |
|  |  | **Scaphocephaly** | **Trigonocephaly** | **Anterior Plagiocephaly** | **Healthy** | | **Sensitivity/ Recall** | |
| **Actual** | **Scaphocephaly** | **684** | 0 | 0 | 0 | | 100.0% | |
|  | **Trigonocephaly** | 0 | **360** | 0 | 0 | | 100.0% | |
|  | **Plagiocephaly** | 0 | 0 | **242** | 1 | | 99.6% | |
|  | **Healthy** | 0 | 0 | 1 | **476** | | 99.8% | |
|  | **Precision** | 100.0% | 100.0% | 99.6% | 99.8% | |  | |
|  | **Specificity** | 100.0% | 100.0% | 99.9% | 99.9% | |  | |

# Table legend

- **Supplementary Table 1**: The confusion matrix of the training sets with computed recall/sensitivity, precision and specificity. Due to the use of the 10-fold cross-validation method, training numbers are 9 times higher than the number of patients.
